# Connecting Biological and Synthetic Approaches for Electrocatalytic CO_2_ Reduction

**DOI:** 10.1002/anie.202310547

**Published:** 2023-12-12

**Authors:** Samuel J. Cobb, Santiago Rodríguez‐Jiménez, Erwin Reisner

**Affiliations:** ^1^ Yusuf Hamied Department of Chemistry University of Cambridge Lensfield Road Cambridge CB2 1EW UK

**Keywords:** Biocatalysis, Electrochemical CO_2_ Reduction, Electrolyzer, Heterogenized Catalysts, Local Environment

## Abstract

Electrocatalytic CO_2_ reduction has developed into a broad field, spanning fundamental studies of enzymatic ‘model’ catalysts to synthetic molecular catalysts and heterogeneous gas diffusion electrodes producing commercially relevant quantities of product. This diversification has resulted in apparent differences and a disconnect between seemingly related approaches when using different types of catalysts. Enzymes possess discrete and well understood active sites that can perform reactions with high selectivity and activities at their thermodynamic limit. Synthetic small molecule catalysts can be designed with desired active site composition but do not yet display enzyme‐like performance. These properties of the biological and small molecule catalysts contrast with heterogeneous materials, which can contain multiple, often poorly understood active sites with distinct reactivity and therefore introducing significant complexity in understanding their activities. As these systems are being better understood and the continuously improving performance of their heterogeneous active sites closes the gap with enzymatic activity, this performance difference between heterogeneous and enzymatic systems begins to close. This convergence removes the barriers between using different types of catalysts and future challenges can be addressed without multiple efforts as a unified picture for the biological‐synthetic catalyst spectrum emerges.

## Graphical abstract

Electrochemical CO_2_ utilization has quickly expanded and diversified into employing a wide range of catalysts and device configurations. This mini‐review seeks to highlight the significant progress and common elements in heterogeneous, small molecule and enzymatic systems to bridge the gap between these seemingly disparate approaches and to provide a more holistic and unified strategy to develop the field of electrocatalytic CO_2_ reduction.

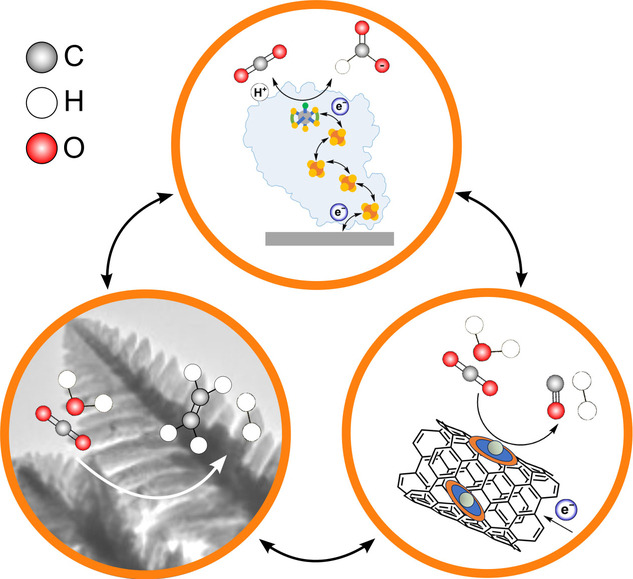

## 1 Introduction

**Figure 1:**
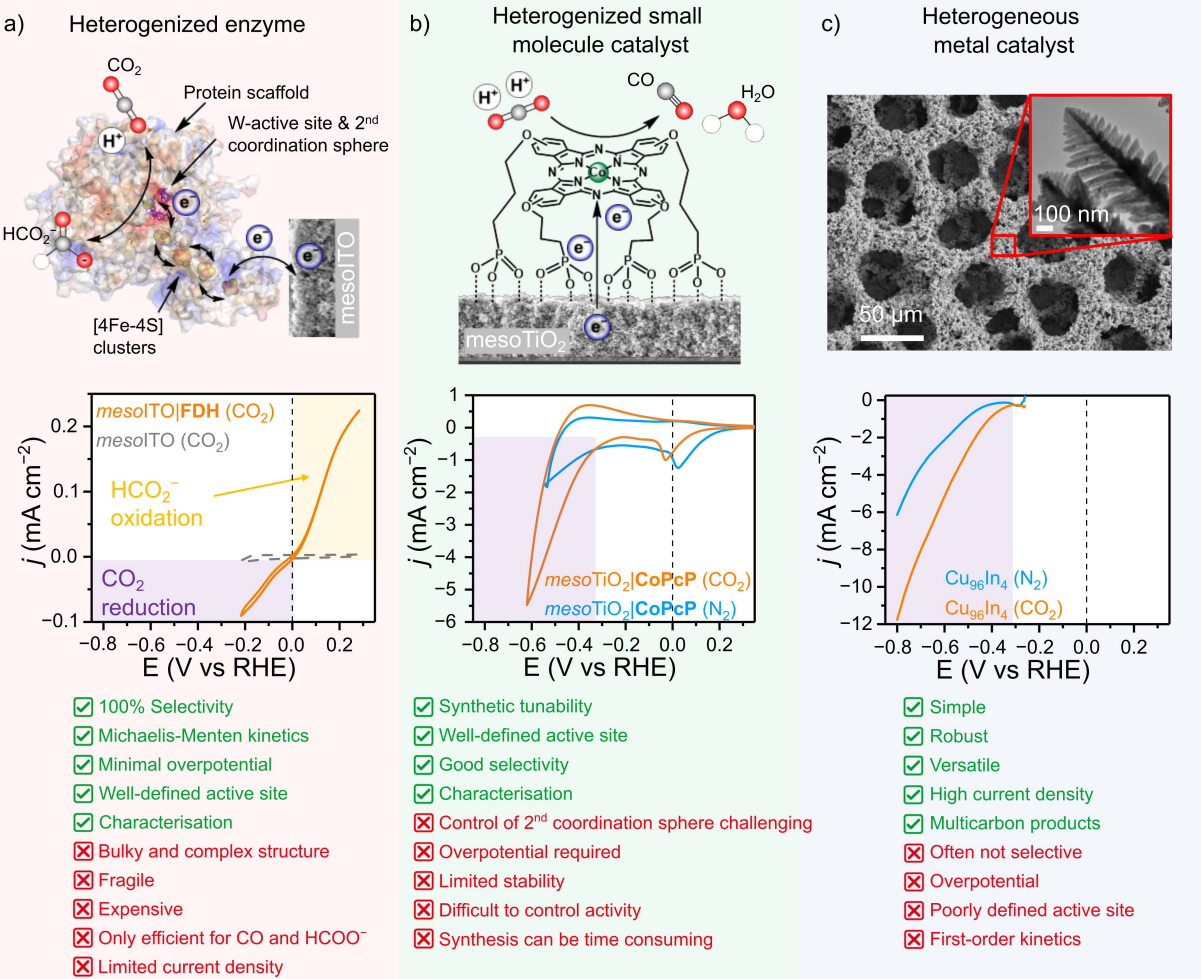
Comparison of biological and synthetic CO_2_R catalysts, their electrocatalytic profile and current advantages and disadvantages for each system. (a) Formate dehydrogenase (FDH, Desulfovibrio Vulgaris Hildenborough structure, PDB: 6SDV)^[19]^ immobilized on a mesoporous ITO electrode (mesoITO),^[6]^ (b) a phosphonate‐containing molecular cobalt phthalocyanine catalyst (CoPcP)^[20]^ immobilized on a mesoporous TiO_2_ electrode (mesoTiO_2_) and (c) scanning electron microscopy and (c, inset) high‐resolution transmission electron microscopy images of a hierarchical bimetallic Cu_96_In_4_ catalyst.^[21]^ The cyclic voltammograms (CVs) of each system highlight their different performance for CO_2_R catalysis. The purple and yellow shaded regions in the CVs highlight the catalytic regions for CO_2_ reduction (a‐c) or formate (HCO_2_^−^) oxidation (a), respectively. The black dashed line at 0 V vs RHE is used as an eye guide to compare the catalytic onset potentials in the CVs.

Electrocatalysis as a means for turning CO_2_ into valuable products provides an opportunity to close the anthropogenic carbon cycle.[^1^, ^2^] This prospect has provided significant motivation for the field to expand rapidly, giving rise to a variety of novel approaches, such as developing electrode heterogenized enzymes, immobilized small molecule catalysts, or heterogeneous metals and alloys as CO_2_ reduction (CO_2_R) catalysts (Figure 1). The ideal electrocatalyst should: capture CO_2_ from dilute streams, be selective for a single product, operate at high rate with low overpotential, and be inexpensive, robust and readily available in large quantities. Although different strategies are being pursued to assemble efficient CO_2_R catalysts, the underlying chemistry and motivation remain the same. However, the different approaches are often pursued in isolation and shared aspects are often overlooked. By taking a holistic approach, it is possible to identify where systems can learn from each other and identify areas where further work is required.

Enzymes provide inspiration as “ideal” catalysts for the development of electrocatalytic systems as they can exhibit exceptionally high activities, often with near unity selectivity and are capable of driving reactions with minimal overpotential, frequently exhibiting catalytic reversibility (in other words, bidirectional catalysis at the thermodynamic potential; see Figure 1a).^[3]^ Crucially, these ideal features arise from their four‐dimensional structural and temporal complexity, where tunnels are gated by structural changes over time to control substrate and product transport.^[4]^ This structure includes a well‐defined catalytic active site, where CO_2_R catalysis takes place, often at metallic centers (Fe, Ni, Mo, W) and buried within the protein scaffold, which allows fast multi‐electron transformations aided by co‐factors. A secondary coordination sphere located around the active site plays a major role in breaking the scaling relationship^[5]^ by binding the substrate through intermolecular interactions to stabilize intermediate species to minimize the overpotential and thus enabling catalytic reversibility (Figure 1a).[^3^, ^6^, ^7^] The outer protein scaffold provides tailored hydrophilic and hydrophobic environments that facilitate the structural alignment over time and orientation of the active site to catalyze a specific substrate resulting in high specificity and selectivity.

The desirable properties of enzymes allow them to serve in some aspects as model catalysts for synthetic CO_2_R systems, but their purification, cost and fragility continue to limit their practical application. Enzymes can be heterogenized onto electrode surfaces, providing a model of the ideal electrocatalyst with a low active site density, compared to heterogenized small molecule and metal catalysts.^[8]^ The heterogenization of enzymes onto an electrode surface introduces its own challenges; as the complex structure that imparts performance, also makes the transfer of electrons to the active site challenging. Enzymes immobilized on electrodes are diametrically opposed to most bulk metal heterogeneous material catalysts (subsequently referred to as heterogeneous catalysts) such as Au (Figure 1) as the nature of the active site in heterogeneous catalysis is structurally simple but often poorly defined. While it is possible to identify areas of locally higher activity, there is no direct comparison to the discrete active site of an enzyme. Between these two extremes lie small molecule catalysts, whose structures can be synthetically designed to allow for the inclusion of metals and bio‐inspired approaches, and the ability to tune the properties of the catalyst.[^9^, ^10^] Small molecule catalysts (synthetic metal complexes) possess a discrete and chemically tuneable active site, but with drastically reduced size and structural complexity compared to enzymes, which generally does not extend beyond the first coordination sphere, with notable exceptions.[^9^, ^11^, ^12^, ^13^, ^14^, ^15^, ^16^, ^17^, ^18^] Thus, small molecule catalysts do not yet match the performance of enzymes.

Researchers working on enzyme, synthetic small molecule and metal catalysts are developing CO_2_ reduction systems at a significant pace, but operate often in separate communities. There are many common challenges where inspiration can be taken from each other to accelerate and to ultimately commercialize electrocatalysts. In our laboratory, we have been studying all three types of catalysts for over a decade and in this minireview we will directly compare and contrast some of the recent progress across the field, without limit to a particular subdiscipline and highlight areas where progress to bridge the gap between these fields has been made.

## 2 Enzymes as Model Electrocatalysts

We focus in this minireview on enzymes that can perform some of the key reactions found in CO_2_R and can take electrons directly from an electrode (direct electron transfer, DET) in a manner analogous to a small molecule catalyst on an electrode, or a discrete active site in bulk heterogeneous catalysis. Natural CO_2_ fixation occurs through two‐electron reductions in pH benign aqueous solution. We discuss the CO_2_ reducing enzymes most relevant for technological applications: Formate dehydrogenases (FDH, for CO_2_R to formate)[^19^, ^22^] and carbon monoxide dehydrogenase (CODH, for CO_2_R to CO).^[23]^ While there are significant insights to be gained from cofactor dependent or mediated enzyme catalysis, these have recently been reviewed extensively elsewhere,^[24]^ with cofactor and mediator recycling offering unique challenges that are not transferable to the DET of heterogeneous and small molecule systems. The structures of FDH and CODH are well resolved, with techniques such as X‐ray crystallography,[^19^, ^23^, ^25^] cryo‐electron microscopy^[26]^ and increasingly computational techniques based on neural networks such as Alphafold,^[27]^ allowing the complete enzyme structure to be elucidated with high resolution, even for different stages of the catalytic cycle. Using techniques such as Infrared, Raman and Electron Paramagnetic Resonance spectroscopy, the properties and mechanisms responsible for the often reported unrivalled selectivity, activity and efficiency of enzymes can be elucidated.^[28]^ This counter‐intuitively results in a level of structural and mechanistic detail for enzymatic catalysis that is not available for the seemingly much simpler heterogeneous systems.

The active sites of CODH and FDHs are remarkably well conserved, with exceedingly similar structural composition and arrangement wherever these enzymes are found, demonstrating nature's ability to converge on an optimal structure. Metal‐dependent FDHs have a Mo[^29^, ^30^] or W[^19^, ^22^, ^31^] metal ion in a bis‐metal‐binding pyranopterin guanine dinucleotide cofactor, which is also coordinated to a cysteine or selenocysteine ligand and a sulfido ligand, whereas NiFe‐CODHs have a Ni‐3Fe‐4S cluster coordinated with 4 cysteines and an additional Fe ion.[^23^, ^32^, ^33^]

The active site of these enzymes is supported by a second coordination sphere that can shape the electronic and hydrogen bonding of the active site, along with allosteric effects. For instance, in the case of W‐FDH the second coordination sphere is composed by two amino acids, one histidine and one arginine adjacent to the W active site.^[34]^ Beyond the second coordination sphere the entire enzyme structure—often >100 kDa—is complicit in conferring its activity and selectivity, their large bulk and complexity acting to drive their unique performance. Many FDHs possess discrete hydrophilic, positively charged[^35^, ^36^] and hydrophobic channels,[^19^, ^36^] for the transport of formate and CO_2_ respectively, whereas CODH has a hydrophobic gas channel to guide the transport of CO_2_ and CO, along with a proton channel.^[23]^ As enzymes are oxidized and reduced, conformational changes and gating are hypothesized to occur, which open and close these channels to facilitate the transport of substrate and product to and from the active site as required.[^19^, ^31^] This control of substrate allows the active site of the enzyme to operate in a local environment that is isolated from the bulk solution and closely tailored to perform efficiently the desired reactions. This environment is thus optimized in both space and time, allowing enzymes to operate as 4D catalysts where their properties change with time throughout the catalytic cycle to diminish kinetic limitations during the reaction mechanism.

The large size of enzymes—arising from the structural features responsible for their optimal performance—introduces challenges in their heterogenization onto surfaces, as only a limited number of orientations place the electrode close enough to the distal co‐factor (e.g. [4Fe‐4S] cluster) for DET to occur. Therefore, they must be orientated correctly on the surface, with the electrode playing the role of the interfacial redox partner, providing electrons to the intra‐protein electron relays and the active site. To solve this challenge that is unique to enzyme catalyzed CO_2_ reduction, electrode engineering is required.[^37^, ^38^, ^39^] Varied approaches have been used to facilitate charge transfer between electrodes and heterogenized enzymes. This extends from the protein film voltammetry approach, originally developed for cytochromes,[^40^, ^41^] of non‐covalently immobilizing FDHs and CODHs on rough carbon surfaces, such as edge plane pyrolytic graphite[^22^, ^42^, ^43^, ^44^, ^45^, ^46^] and graphite epoxy,[^47^, ^48^] to engineered non‐covalent DET. For instance, mimicking the surface charge of a natural redox partner[^37^, ^38^] or including moieties such as adamantane onto the electrode surface that can interact with hydrophobic patches on the enzyme.^[49]^ Furthermore, stable DET to an electrode can be facilitated through His‐tags on the enzyme^[50]^ and covalent binding.^[51]^

Structured 3‐dimensional (3D) electrodes, for instance porous metal oxide electrodes[^6^, ^52^, ^53^, ^54^, ^55^] can be used to increase the geometrical enzyme loading due to their high specific surface area, and facilitate stable immobilization in an electroactive orientation due to multi‐point contact.[^39^, ^52^] Redox polymers[^56^, ^57^, ^58^, ^59^, ^60^] also offer increased enzyme loading, whilst also facilitating electron transfer to enzymes,[^56^, ^57^, ^58^] and protecting from deactivation by O_2_—a typical inhibitor for dehydrogenases.[^56^, ^59^, ^60^, ^61^] The large footprint of enzymes (on the order of 10–100 nm^2^) compared to small molecule and heterogeneous catalysts limits the absolute number of active sites and therefore current densities, despite the high turnover frequencies of the catalyst that partly compensate for low catalyst loadings. However, when the enzyme‐electrode interaction is optimal to allow DET, the highly active reversible catalysis of CO_2_R is possible which is an ultimate goal of synthetic CO_2_R (Figure 1).^[30]^

## 3 Metal Complexes as Tuneable Catalysts

**Figure 2:**
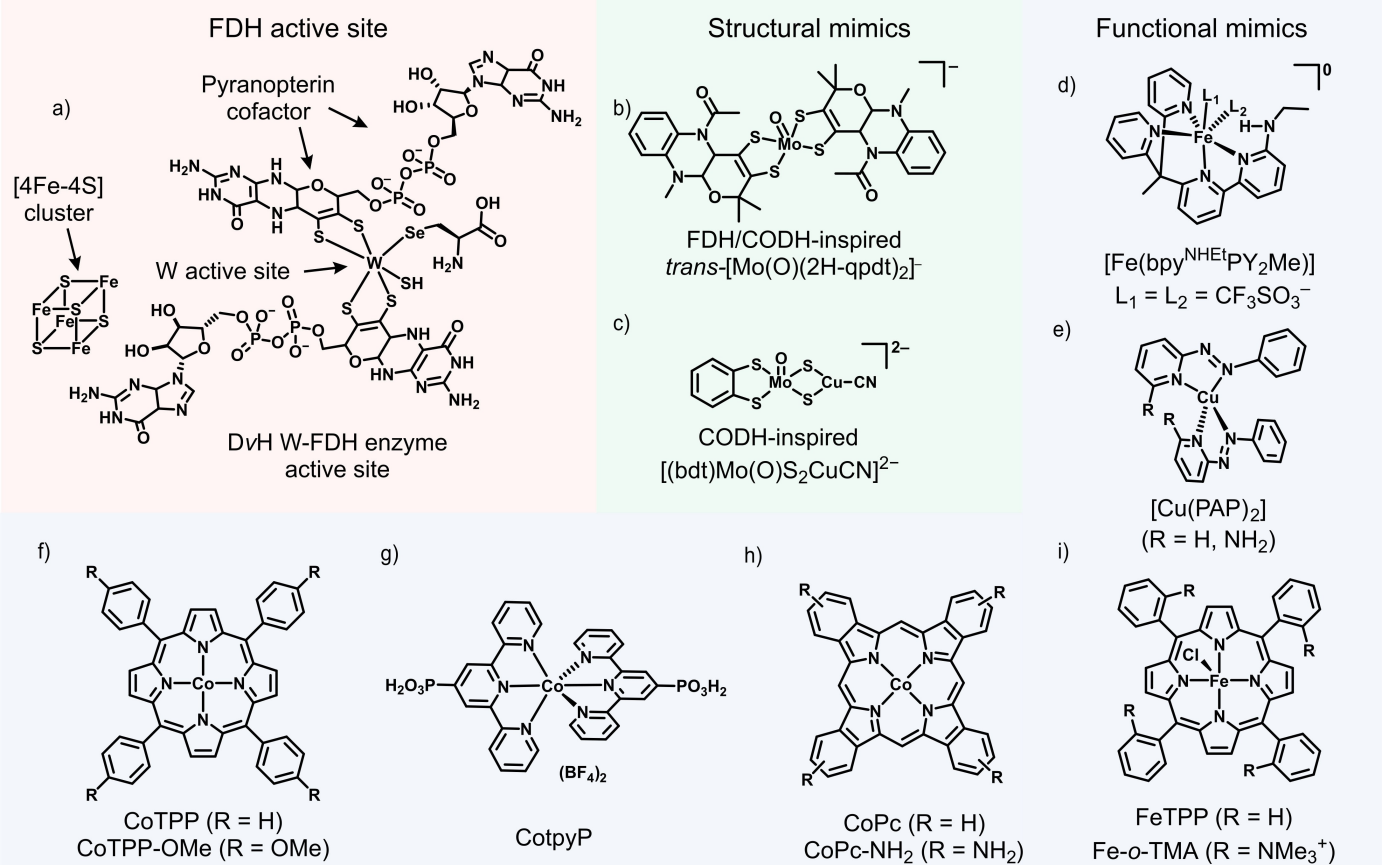
Structures of FDH enzyme's active site and small molecule catalysts: (a) active site of *Desulfovibrio vulgaris* Hildenborough (*Dv*H) W‐containing formate dehydrogenase (W‐FDH), (b) FDH/CODH‐inspired trans‐[Mo(O)(2H‐qpdt)_2_]^−^,^[87]^ (c) CODH‐inspired [(bdt)Mo(O)S_2_CuCN]^2−^,^[63]^ (d) CODH‐inspired [Fe(bpy^NHEt^PY_2_Me)],^[15]^ (e) CODH‐inspired [Cu(phenylazopyridine)_2_],^[88]^ (f) cobalt porphyrins,[^72^, ^89^] (g) phosphonate‐containing cobalt terpyridine,^[90]^ (h) cobalt phthalocyanines,[^91^, ^92^, ^93^, ^94^] and (i) iron porphyrins.^[16]^

From a reductionist perspective, stripping away the protein shell apart from the first and second coordination sphere from an enzyme should provide the target structure in biomimetic chemistry (Figures 1a and 2a–e). Although precise structural biomimics of FDH and CODH are not yet synthetically available, the inspiration derived from the active sites of enzymes fuels the preparation of bioinspired small molecule catalysts (Figures 2b–e).[^6^, ^15^, ^62^, ^63^] The development of small molecule systems for CO_2_R is a strong and topical research area, annually yielding hundreds of new systems either operating in homogeneous conditions or, more recently, heterogenized onto electrodes.[^10^, ^15^, ^64^, ^65^, ^66^, ^67^, ^68^]

For heterogenized small molecule catalysts, their size means the distance between the active site and the electrode surface is within tunnelling distance, making electron transfer straightforward. However, the presence of an interface or spacer between the catalyst molecule and the electrode surface can introduce stability and activity limitations originating from catalyst immobilization. Heterogenized small molecule catalysts commonly contain a linker moiety optimized for a stable and irreversible immobilization that must be tuned to the electrode surface chemistry. This immobilization can be adsorption based to carbon through non‐specific physisorption,^[69]^ π‐π interactions[^20^, ^70^, ^71^, ^72^, ^73^] or polymerisation onto the carbon surface.[^74^, ^75^] Covalent interactions are also possible—for instance diazonium grafting to carbon, the formation of phosphonate esters by phosphonic acid groups on metal oxide surfaces^[76]^ or Au‐thiol bonds.^[77]^ It is also possible to incorporate small molecule catalysts into conductive structures such as metal–organic frameworks.^[78]^

Immobilization can also facilitate efficient electron transfer, when a small molecule catalyst is covalently coupled to a surface through a conjugated system, the electronic structure of the catalyst is shown to be coupled to that of the electrode material. This has a significant effect on the catalytic mechanism, acting comparably to the active site in heterogeneous catalysis, but with a well‐defined structure.^[79]^ The nanometer size of small molecule catalysts can also complicate the incorporation of enzyme‐like features such as second and outer coordination spheres. Despite that, tuning the electrode surface chemistry (see below) can provide enzyme‐like characteristics to heterogenized molecular sites, leading to improved efficiency and selectivity.^[80]^ Moreover, electrode heterogenization of metal complexes through non‐covalent (e.g. π‐π stacking or electrostatic immobilization) or covalent immobilization can decrease side reactions and improve electronic coupling.^[81]^ This can also result in higher chemical stability, lower overpotentials and potentially different product selectivity compared to homogeneous molecular systems,[^72^, ^82^, ^83^, ^84^, ^85^, ^86^] as observed for cobalt tetraphenylporphyrins and phosphonated cobalt bisterpyridine catalysts (Figures 2f,g) immobilized on carbon nanotubes or metal oxide surfaces, respectively. Efforts to understand the effects arising from molecule‐surface interactions are ongoing,^[95]^ and increasing our understanding of these phenomena could significantly enhance our ability to develop improved synthetic molecular systems for CO_2_ reduction and beyond. Heterogenized metal complex catalysts have shown close to unity selectivity for CO_2_R to CO[^72^, ^80^, ^85^] and formate,^[90]^ and accessed other complex reduction products such as methanol (40 % selectivity) and methylamine (10 % selectivity)[^91^, ^92^] (Figure 2f–i).

An important feature unique to small molecule catalysts is that their active site can be tuned readily and accurately through chemical modifications. Such versatility makes them great model systems to develop structure‐activity relationships and gain mechanistic insights; an approach not easily possible with enzyme or heterogeneous electrocatalysts. However, while small molecule catalysts are structurally a step closer towards enzymes, they still lack the enzyme's high CO_2_ affinity, low overpotential and catalytic rate (Figures 1a,b). Efforts towards closely mimicking CO_2_R enzymatic active sites have been proven synthetically challenging, and the activity and overpotential requirements of the resulting discrete biomimetic small molecule catalysts were worse than those of heterogenized enzymes.[^15^, ^62^, ^63^, ^87^, ^96^, ^97^, ^98^, ^99^, ^100^] For instance, FDH‐inspired *trans‐*[Mo(O)(2H‐qpdt)_2_]^−^ (Figure 2b) and its *cis* isomer^[87]^ and CODH‐inspired [(bdt)Mo(O)S_2_CuCN]^2−^ (Figure 2c) could electroreduce CO_2_ to formate with ≈39 and 70 % Faradaic efficiency (FE) and exhibited a turnover frequency of 5 and 2 h^−1^ in acetonitrile, respectively.[^63^, ^87^] For comparison, W‐FDH could convert CO_2_ to formate with 100 % FE and turnover frequencies of 1000 s^−1^ in solution assays^[19]^ and ≈50 s^−1^ on electrodes^[101]^ in aqueous solutions. Nevertheless, the secondary coordination sphere interactions found in other enzymes such as H_2_‐producing hydrogenase have been successfully bio‐mimicked resulting in reversible H_2_ cycling small molecule catalysts.^[102]^ These examples further highlight the potential and crucial role that supramolecular environments (e.g. second coordination sphere and 3D protein scaffolds) play around an active site, and the existing potential to develop improved biomimetic CO_2_ reduction catalysts.

The introduction of a second coordination sphere in CO_2_R small molecule catalysts via synthetic means is challenging but possible: recent examples are based on iron polypyridine and porphyrin catalysts (Figures 2d,i).[^15^, ^16^] Incorporation of a Lewis acidic moiety near the catalytic center can mimic some of the key features of the enzymatic active site in NiFe‐CODH enzymes and this strategy has demonstrated to benefit catalytic activity and CO selectivity in iron polypyridine complexes, and even enable catalytic CO_2_/CO bidirectionality in copper (phenylazo)pyridine complexes (Figure 2e).[^15^, ^88^] The integration of cationic groups in iron porphyrins demonstrated that proximal coulombic interactions stabilized the negatively charged Fe‐CO_2_ intermediates, affording much lower overpotentials (220 vs ≈800 mV) and high CO turnover frequencies (>300 s^−1^) compared to the unfunctionalized analogues (Figure 2i).^[16]^

Beyond these examples, there are exciting opportunities to enhance small molecule catalysts’ performance: (i) by incorporating enzyme‐like features, e.g. using concerted proton‐electron transfer mediators to improve metal‐hydride formation and CO_2_R rates,^[103]^ or integrating enzyme‐like multimetallic active sites in polyanionic clusters,^[104]^ or encapsulating small molecule catalysts within polymeric matrices[^105^, ^106^, ^107^, ^108^, ^109^] or protein scaffolds.[^110^, ^111^] These systems can leverage supramolecular environments (e.g. hydrophobic/hydrophilic channels) to tune overpotential and product selectivity while also improving immobilization and O_2_ protection; (ii) covalently heterogenizing small molecule catalysts into graphitic materials to eliminate redox intermediates and to access high reaction rates at low overpotentials.^[79]^

## 4 Heterogeneous Materials as Scalable Catalysts

Heterogeneous catalysts, in contrast to (bio)molecular systems offer simplicity, in particular in preparation as the electrode is a bulk material that is catalytically active. As the catalyst and electrode surface are intrinsically linked, electron transfer is straightforward, and the immobilization challenges encountered for (bio)molecular systems are avoided. However, heterogeneous CO_2_R does have significant limitations in its performance in terms of selectivity and energy efficiency, requiring commonly a large overpotential. In heterogeneous CO_2_R, the active site is not often immediately apparent as a discrete site that can be studied. There have been some recent efforts to produce well‐defined heterogeneous single atom catalysts that sit between traditional heterogeneous materials and small molecule catalysts that have been extensively reviewed elsewhere.[^112^, ^113^]

In most cases, the activity can be related to the crystal structure of the material, with different crystal facets demonstrating varied activities for CO_2_R. For instance, from single crystal Au studies,^[114]^ flat Au(100) facets have higher coordinated surfaces proving significantly (≈20×) less active for CO_2_R than less coordinated ones such as Au(110) and the steps of Au(211). The hydrogen evolution reaction (HER) is almost independent of crystal face, and makes the low coordination facets such as Au(211) more likely to display higher FE for CO_2_R products due to their relatively higher CO_2_R rate. Thus, unlike enzymatic CO_2_R where the secondary structure imparts active site selectivity, the same active sites on Au are in competition for HER and CO_2_R, with CO_2_ being energetically favored.^[115]^ While structure–activity relationships based on crystal facets are useful to design more efficient heterogeneous CO_2_R catalysts comparisons to enzymatic catalysts are challenging, as bulk crystal structure bears no relation to the well‐defined supramolecular complex in the active site of an enzyme. A point highlighted by the inactivity of the metal centres in FDH (W and Mo) and CODH (Ni and Fe) for CO_2_R in their metallic forms,^[116]^ although some CO_2_R activity has been observed for Fe_4.5_Ni_4.5_S_8_ minerals, the composition of which can be considered to be bioinspired.[^117^, ^118^]

This situation is further complicated as for the majority of heterogeneous CO_2_R studies, the electrode is not a single crystal surface, and is instead a polycrystalline or nanostructured material made of a combination of different crystal facets of different activity, the combination of which is heavily affected by electrode preparation. Furthermore, the boundaries where these grains meet and their accompanying surface dislocations can have a significant contribution to the CO_2_R activity and can dominate the observed bulk response.[^119^, ^120^] While the active site is not defined in the same sense as for a (bio)molecular catalyst, it has been shown to be possible to limit CO_2_R by selectively blocking steps and under‐coordinated defect sites using Pb under potential deposition.^[114]^ Comparisons can be drawn to inhibition studies on an enzyme, where a ligand such as azide can be used to block the active site of formate dehydrogenase on an electrode.^[47]^

While Au has been discussed predominantly above, the challenges are similar for other heterogeneous CO_2_R materials such as Ag, Pb, Bi and Sn. Cu sits apart due to its ability to produce a more complex array of products. While this can be advantageous as Cu can produce a range of higher order products including: CH_4_, alcohols and C_2+_ products—something that a single FDH or CODH enzyme alone cannot, with nature typically reliant on multiple 2e^−^ reductions in cascades—it also adds another degree of complexity as the material structure and facet composition can reconstruct under electrochemical conditions which can critically affect the product distribution.[^121^, ^122^]

Recently, in a nod to the second coordination sphere of an enzyme, self‐assembled monolayers have demonstrated the ability to significantly alter the product selectivity of Cu. Such approaches offer the possibility to break scaling relationships[^123^, ^124^]—in a similar way to how the second coordination sphere makes catalytically poor metals such as W in FDH and Ni and Fe in CODH into reversible catalysts. Hydrophobic layers can increase the selectivity of C_2_ products such as ethene^[125]^ by trapping CO_2_ at the electrode surface, while surface adsorbed 4‐mercaptopyridine increased the FE for formate, supressing CO and C_2+_ production while H_2_ production was unaffected.^[126]^ Density Functional Theory calculations indicated that the immobilized 4‐mercaptopyridine destabilized the intermediates responsible for the formation of CO, which also acts as the initial step for the formation of C_2+_ products. While in this system the exact molecular identity of the active site is not known, it has been possible to understand some of the interactions between the active metal (Cu) and the self‐assembled monolayer (loosely analogous to pendant amino acid ligands around the metal active site in an enzyme). This takes a step towards (bio)molecular catalysts while retaining many of the properties of heterogeneous catalysis.

## 5 Local Environments in CO_2_R Catalysis

**Figure 3:**
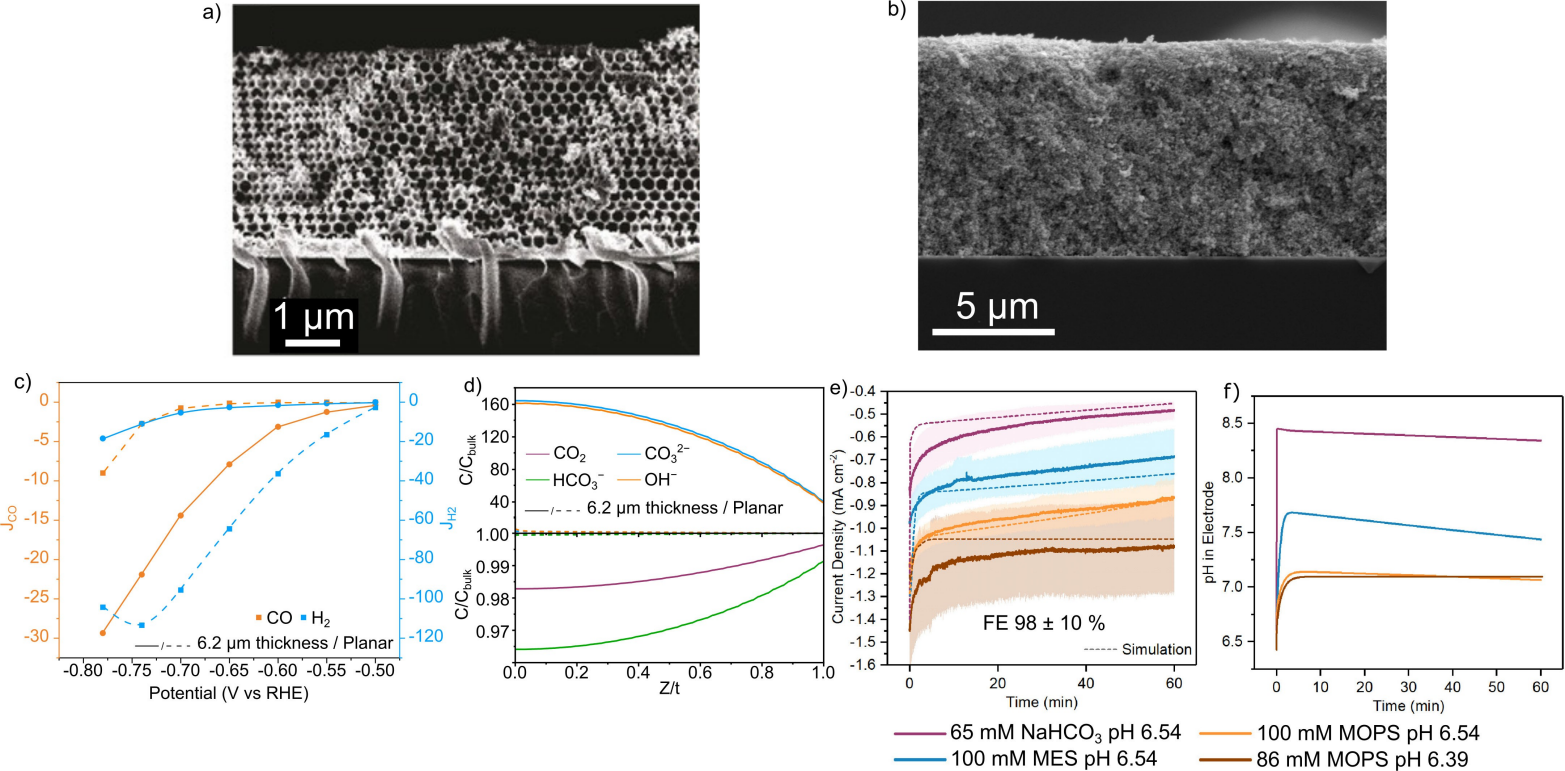
Electrodes and electrolyte design influences the local environment of catalysis. (a) Inverse opal porous Ag electrode^[128]^ Reproduced from Ref. [128] Copyright (2016) with permission from John Wiley and Sons/Wiley VCH. (b) Mesoporous ITO electrode for enzyme immobilization. Reproduced from Ref. [101], Copyright (2022) with permission from the authors. (c) Experimental and simulated current densities for HER (blue) and CO_2_R (orange) in an inverse opal Ag electrodes of 0 μm (planar, dashed lines) and 6.2 μm thickness (solid lines).[^128^, ^129^] (d) Local concentrations of CO_2_ (purple), HCO_3_^−^ (brown), CO_3_^2−^ (blue) and OH^−^ (orange) within inverse opal Ag electrodes of 0 μm (dashed lines) and 6.2 μm (solid lines), X axis is normalized distance from/within the electrode where 0 is the back of the electrode and 1 is the solution exposed face.^[129]^ (e) Dependence of current density on electrolyte composition for CO_2_R by W‐FDH immobilized on mesoporous ITO,^[101]^ (f) pH within a W‐FDH immobilized mesoporous ITO electrode under catalysis in a range of electrolytes (65 mM NaHCO_3_ buffer (purple), 100 mM 2‐(N‐morpholino)ethanesulfonic acid (MES) (blue), 100 mM 3‐(N‐morpholine)propanesulfonic acid (MOPs) (orange), 86 mM MOPS (brown)).^[101]^

To produce high current densities in an electrolysis system (electrolyzer), large amounts of a highly efficient catalyst must be electronically coupled to an electrode surface. The differing properties of catalysts require a range of approaches due to their varied limiting factors and footprints. Consistent across systems is the desire to access the z dimension and move from 2D to 3D architectures through the use of porous electrodes, which offer an increase in practically relevant geometric (albeit not volumetric/gravimetric) current densities and the opportunity to tune properties such as the local environment (Figure 3a,b).[^127^, ^128^, ^129^] For heterogeneous catalysis, a 3D design is intrinsically linked to increase in active sites and catalytic rates, but for enzymatic and heterogenized small molecule catalysts the current density is proportional to the amount of catalyst immobilized where an increased surface area offers the potential for more catalyst loading for increased geometric current densities.^[52]^ Bespoke 3D architectures have also enabled advantages for enzymes such as protection from denaturation, improved electrical connection and improved cofactor recycling.[^130^, ^131^, ^132^, ^133^] The overall performance of an electrocatalytic system extends beyond the catalyst and its coordination sphere, with the supply of electrons, protons and substrate, or product removal all offering the potential to act as a bottleneck that can define the performance of a system. The surface confined nature of catalysis (Figure 3) means that these mass transport processes create a local environment that is markedly different to that in a bulk solution.

**Figure 4:**
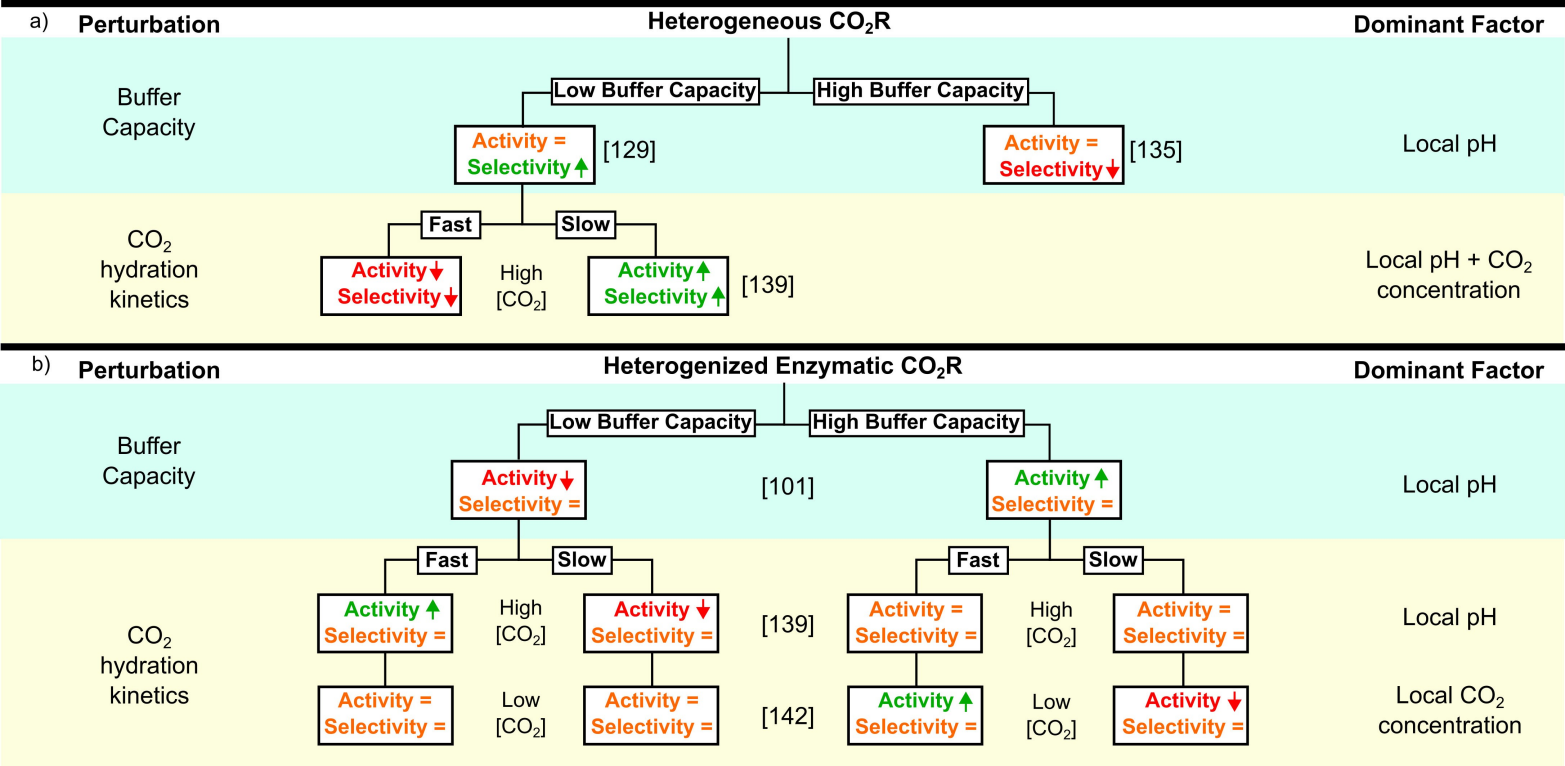
Effect of perturbations in the solution properties—in particular buffer capacity and CO_2_ hydration kinetics. (a) Effect on the performance of heterogeneous catalysis, driven by the lack of selectivity. (b) Effect of buffer capacity and CO_2_ hydration kinetics on enzymatic CO_2_R in terms of selectivity and activity, where enzymes are 100 % selective and display a pH‐dependent activity, demonstrating the contrasting response of heterogeneous catalysis to the same perturbations (a). The dominant factor defining the response of the system is dependent on the catalyst properties. Square brackets indicate literature source.

Porous electrodes of heterogeneous CO_2_R catalysts such as Ag (Figure 3a),^[128]^ Au,^[127]^ and Cu^[134]^ have been reported, with a primary motivation of creating a local environment to suppress side reactions such as HER (Figure 3c,d). A basic local pH is formed within the electrode and the pH becomes more basic during CO_2_R, which has been observed spectroscopically on planar electrodes,^[135]^ although spectroscopy within porous electrodes still proves experimentally challenging.^[136]^ Recently, confocal microscopy with photoacids has emerged as a viable technique for measuring the pOH (‐log_10_[OH^−^], equivalent to pH for OH^−^ ions) within porous gas diffusion layers.[^137^, ^138^] With Ag electrodes an increase in thickness of the porous electrode from 0.5 to 2.7 μm increased the FE from 5 to 80 % at −0.7 V vs RHE (Figure 3c). This was postulated, and later shown by multiphysics modelling to be due to the local pH within the electrode acting to suppress H_2_ evolution within the porous structure (Figure 3d).^[129]^ This strategy can also increase the amount of C_2+_ products formed on a Cu electrode.^[134]^ For this reason, high buffer capacity electrolytes have been shown to increase HER and lower the FE due to a more acidic local pH (Figure 4).^[135]^ The local environment created within a porous electrode in heterogeneous catalysis can, in a way, be considered to be a poor surrogate for the environment created by the protein structure around the active site of the enzyme. Where the enzyme structure acts to control the access of substrate (CO_2_ and H^+^) and the removal of products, the hindered diffusion in a porous heterogeneous catalyst serves an analogous role.

Enzymatic CO_2_R is not immune to the creation of local pH environments in porous electrodes; but its selectivity is irrespective of pH, meaning a basic local pH does not confer the selectivity advantages observed in heterogeneous CO_2_R. Instead, the local pH should be optimized to match proton availability to maximize catalytic activity of the enzyme for CO_2_R through the judicious choice of the buffer system (Figure 3e,f).^[101]^ Generally a buffer with high buffering capacity is beneficial compared to an insufficiently buffered NaHCO_3_ solution (Figure 4b). For FDH from *Desulfovibrio Vulgaris* Hildenborough in porous metal oxide electrodes, Good's buffers were used to match the local pH to the optimal of the enzyme (pH=7.1) using multiphysics modelling.^[101]^

The additional hydration step of converting CO_2_ to H_2_CO_3_ before deprotonation to HCO_3_^−^ (apparent pK_a_ of CO_2_/HCO_3_^−^=6.34) introduces a kinetic barrier that slows the rate of equilibration, allowing for large pH changes at even modest current densities (>1 pH unit for ≈10 μA cm^−2^),^[139]^ which can suppress HER in heterogeneous systems as the buffer system is not kinetically fast enough to counteract the increase in pH (Figure 4a).[^127^, ^128^, ^139^, ^140^] However, the demonstrated unfavorability of basic local pH environments for enzyme systems when using a poorly buffering system such as bicarbonate, means that conceptually these slow kinetics do not offer an advantage (Figure 4b).^[101]^ On the other hand, it has been demonstrated that the co‐immobilization of an additional enzyme, carbonic anhydrase (CA), to catalyze CO_2_ hydration^[141]^ improves the activity of CO_2_ reduction by FDH, with no effect on selectivity (100 % in all cases) due to a decrease in the local pH closer to the enzyme's optimal.^[139]^ Whereas for heterogeneous CO_2_R on Au, the addition of CA increases H_2_ evolution and decreases CO_2_R, significantly affecting the FE. The differences between enzymatic and heterogeneous CO_2_R become more pronounced when the CO_2_ concentration is considered, as the conversion of CO_2_ into HCO_3_^−^ and H^+^ to mitigate the pH change decreases the local CO_2_ concentration and increases the local HCO_3_^−^ concentration.

The Michaelis–Menten kinetics of enzymes means that the FDH activity is almost unaffected by the reduction in local CO_2_ concentration well above the Michaelis–Menten constant (K_M_), whereas with heterogeneous electrodes CO_2_R follows first order kinetics which leads to a direct decrease in CO_2_R activity. Furthermore, FDH has been shown to only use CO_2_ as a substrate,^[44]^ whereas HCO_3_^−^ can react, for instance, at a Au surface, being the major proton donor for HER. This leads to a decrease in the CO_2_R FE in heterogeneous systems for 3 reasons: 1) reduced local pH, 2) reduced local CO_2_ concentration, and 3) increased local HCO_3_^−^ concentration, whereas enzymes are positively affected by the reduced local pH while all other factors have a negligible effect, further highlighting the differing design considerations from enzymatic to heterogeneous CO_2_R.

FDH co‐immobilized with CA has recently been used at low (10 % to 420 ppm) CO_2_ concentrations.^[142]^ Under these conditions, the CO_2_ concentration does affect the system activity and in KHCO_3_ solutions CA immobilization improved the local pH, but this was counteracted by a lower local CO_2_ concentration (Figure 4b). Adding 3‐(N‐morpholine)propanesulfonic acid (MOPS) buffer to increase the buffer capacity reduced the local pH change, and increased the current density for CO_2_R when CA was co‐immobilized as CO_2_ converted to formate at the electrode surface was rapidly replenished from HCO_3_^−^ increasing the local CO_2_ concentration. This approach enabled direct atmospheric CO_2_R by FDH, reliant on the high selectivity and Michaelis–Menten kinetics of enzymes. Under low CO_2_ concentration, the activity of small molecule catalysts are in‐between that of heterogeneous and enzyme systems.^[143]^

## 6 Implications for Electrolyzers

CO_2_R electrolyzers have been a recent area of interest in CO_2_ reduction due to their potential technological importance, primarily utilizing heterogeneous catalysts, and more recently small molecule catalysts immobilized on gas diffusion electrodes (GDE).[^80^, ^93^, ^144^, ^145^, ^146^, ^147^] This field is growing fast with record performances reported frequently and the emergence of start‐up companies. The main motivation for using GDEs is the ability of these architectures to use fast gas/liquid flow to create a triphasic interface at the catalyst surface, achieving very high CO_2_ local concentrations and mass transport. This enables high current densities compared to standard bulk electrolysis.^[145]^

**Figure 5:**
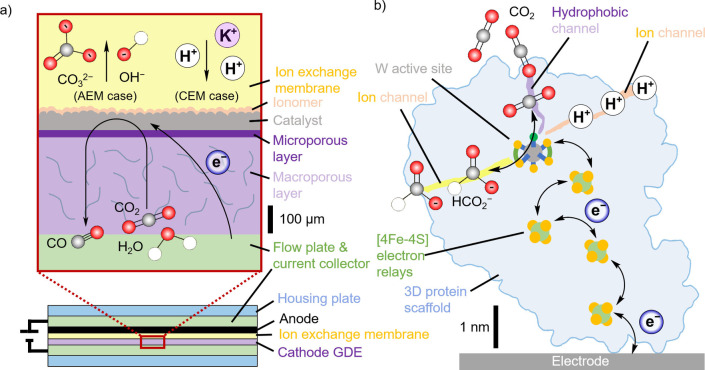
Schematic representation of (a) a CO_2_R electrolyzer highlighting key parts, CO_2_ to CO reaction and ion migration;[^148^, ^156^] and (b) a schematic W‐FDH enzyme immobilized on an electrode, highlighting substrate and product and ion channels, and electron transport pathways.^[36]^ By using the same color code in (a) and (b) we aim to highlight the analogous roles that each component plays (e.g. catalyst and W active site are colored grey). In (a), potassium and proton ions are represented as positively charged purple and white spheres, respectively. CEM and AEM stand respectively for cation and anion exchange membranes. In (b), the two colors used for the ion channel, colored yellow and salmon, highlight its functional ambivalence with respect to the ion exchange membrane (yellow) and ionomer layer (salmon) in (a).

The architecture of CO_2_R electrolyzers generally consists of flow and housing plates that sandwich the anode, polymer electrolyte membrane and cathode GDE (whose role is analogous to that of the outer protein scaffold, [4Fe‐4S] electron relays and ion transport channels in W‐FDH; Figure 5). Other device architectures follow the same principle, but enable catholyte to flow between the membrane and GDE.^[145]^ These architectures allow tuning of important parameters to build high performing CO_2_R electrolyzers. The structure of the GDE cathode normally consists of a macroporous and microporous layer that serve, respectively, as gas diffusion layer and hydrophobic surface (which behave analogously to the hydrophobic channels in enzymes).^[125]^ The catalyst layer is deposited on top of the latter with other additives that can modulate the chemical microenvironment around the catalyst (analogously to the active site and secondary coordination sphere in enzymes).[^148^, ^149^, ^150^]

The catalyst composition, its nanostructure and hydration capacity are important features that can impact product distribution, stability and current density (Table 1).[^147^, ^151^] For instance, the use of molecular hydrophobic/hydrophilic promoters such as 1‐octadecanethiol^[125]^ and 4‐mercaptopyridine,^[126]^ perfluorosulfonic acid and imidazolium ionomers,^[149]^ or co‐loading of hydrophobic PTFE can radically affect the product distribution (HCOO^−^ vs C_2_H_4_) and current densities, which can reach industrially relevant levels (0.2–1 A cm^−2^) with heterogeneous catalysts such as Ag and Cu^[145]^ or small molecule catalysts such as Co phthalocyanines (0.1–0.4 A cm^−2^ with 30–90 % CO FE).[^80^, ^93^] Different membranes such as bipolar membranes, anion and cation exchange membranes,[^149^, ^152^] or porous solid electrolyte,^[153]^ can be used to improve CO_2_R performance. Their choice and thickness can have an impact on ion conductivity (H^+^, Na^+^, K^+^, OH^−^), carbon crossover (HCO_3_^−^/CO_3_^2−^) and water transport between cathode and anode electrodes, and importantly affect product selectivity.^[149]^

**Table 1:** Summary of selected electrolyzer systems employing: metal catalysts (metal), small molecule catalysts and enzymes. For experimental conditions see associated references. j_total_ is the total current density measured.

Type	Catalyst	*J_total_* (mA cm^−2^)	Major Product	Faradaic Efficiency (%)	Stability (h)	Ref.
Metal catalysts	Cu^[a]^	−100 −300	HCOO^−^ HCOO^−^	81 72	6	[126]
	Cu^[b]^	−1.3×10^3^	C_2_H_4_	65–75	60	[145]
	Zn_0.83_Ag_0.17_^[c]^	−100 −500	CO CO	>96 >85	100	[147]
Small molecules	CoPc^[d]^	−400	CO	≈75	5	[80]
	EtO_8_‐CoPc^[e]^	−150 −400	CO CO	≥90 ≈90	24	[80]
	[Ni(CycR)]^2+[f]^	−100	CO	>30	<1	[144]
Enzymes	FDH^[a]^	−18	HCOO^−^	100	5	[154]
	FDH^[b]^	−0.3	HCOO^−^	100	45	[58]
	CODH^[c]^	−4.2 +1.5	CO CO_2_	100 100	1	[49]

[a] Cu co‐immobilized with 15 nmol cm^−2^ of bound 4‐mercaptopyridine; stability given for *j*=−300 mA cm^−2^; 3‐electrode setup. [b] 3.33 mg cm^−2^, co‐immobilized with perfluorinated sulfonic acid on a PTFE support; 3‐ and 2‐electrode setups. [c] 75 μm layer on a GDE; stability given for *j*=−100 mA cm^−2^; 2‐electrode setup. [d] CoPc stands for cobalt phthalocyanine; 9.37 nmol cm^−2^ on carbon nanoparticles; 3‐electrode setup. [e] EtO_8_‐CoPc stands for cobalt octaethoxyphthalocyanine; 9.46 nmol cm^−2^ on carbon nanoparticles; 3‐electrode setup. [f] Cyc stands for cyclam, R=COOH; 1 mg cm^−2^ on GDE; 2‐electrode setup. [g] FDH stands for formate dehydrogenase; 1.2 nmol on PFTE‐coated Ketjen Black; 3‐electrode setup. [h] 0.7 nmol immobilized on methyl viologen‐containing redox polymers; 3‐electrode setup. [i] CODH stands for carbon monoxide dehydrogenase; 52 pmol cm^−2^, immobilized via Van der Waals interactions onto GDE/MWCNTs modified with adamantly groups; 3‐electrode setup.

The use of enzymes in CO_2_R electrolyzers is also emerging (Table 1).[^49^, ^58^, ^154^, ^155^] For instance, a FDH from *Methylbacterium extorquens*, which required an electron mediator, was immobilized on PTFE‐coated Ketjen Black and electroreduced CO_2_ to formate at a low overpotential (when considering only the cathode potential) for 5 h.^[154]^ While this biohybrid system achieved a record current density (−18 mA cm^−2^) using a high enzyme loading (1.2 nmol) on the GDE, the turnover frequency was comparable with solution‐phase enzyme systems (≈170 s^−1^ for a GDE^[154]^ versus ≈50 s^−1^ for solution phase^[101]^). An alternative approach to immobilize FDH to GDEs is the use of redox polymers containing methyl viologen moieties.^[58]^ In this case, the enzyme loading and current density were lower (0.7 nmol, −0.3 mA cm^−2^) but the polymers played a two‐fold role, wiring and protecting the enzymes which were able to operate stably for as long as 45 h.^[58]^ CODH could also be immobilized via van der Waals interactions onto a GDE/MWCNT electrode modified with adamantyl groups.^[49]^ This enzymatic electrolyzer was able to reduce CO_2_ selectively to CO for 1 h at high turnover frequencies (420 s^−1^) and a current density of −4.2 mA cm^−2^ at low overpotential (180 mV). This current was only 30 % higher compared to solution electrochemistry using the same enzyme (−4.2 mA cm^−2^ vs −2.9 mA cm^−2^). Exploiting the catalytic reversibility of this enzyme, the same biohybrid electrolyzer was also able to oxidize CO selectively to CO_2_ at turnover frequencies of 150 s^−1^ (1.5 mA cm^−2^ at 250 mV overpotential).^[49]^

The modest performance of enzymatic CO_2_R electrolyzers highlights that the advantages experienced on heterogeneous and small molecule catalyst systems are difficult to transfer to such biohybrid systems. This is attributed to the superior structural design of the enzymes, which allows them to have extremely high affinity to a substrate (i.e. Michaelis–Menten constant), unity selectivity and operate at the thermodynamic potential. Hence, enzymes are already optimized and the systems and conceptual level advantages of electrolyzers benefit synthetic catalysts more than biocatalysts. Thus, it could be argued that enzymes already possess the attributes GDEs and electrolyzers can introduce to heterogeneous and small molecule catalysts (Figure 5). We suggest that the development of electrolyzers is inadvertently emulating what nature has already perfected. Enzymes, such as FDH, contain the essential features and components necessary for catalysis, i.e., an outer and inner structure where proton, substrate and electron transport and active site environment are perfectly controlled. Therefore, enzymes could be described as the smallest, most efficient electrolyzers, representing the epitome of miniaturized electrolysis systems at the nano‐scale. Thus, by reimagining CO_2_R electrolyzers as highly tuneable enzyme bio‐mimic environments, researchers may consider alternative bio‐inspired solutions to tackle current challenges, such as low energy efficiencies, high overpotentials, low product selectivity, and carbon migration. Considering these systems together can motivate future directions of the field as enzymatic and heterogeneous CO_2_R coalesce, in particular as GDEs may offer also other advantages for enzymes such as stability and robustness that extend beyond pure activity considerations.

## 7 Conclusion

Electrochemical CO_2_ reduction is an area of exceedingly active and vibrant research, with the field diversifying to pursue a range of approaches from enzymatic to synthetic small molecule and heterogeneous catalysis in a broad range of device architectures. While these catalysts perform similar chemistry, their nature and characteristics are highly varied, and each introduce their own advantages and challenges. Enzymatic CO_2_R catalysts already possess many of the properties of an “ideal” model catalyst, due to its multiple coordination spheres controlling the environment around the active site, electron transfer and the mobility of protons, substrate and product. Heterogeneous and small molecule catalysts do not yet possess this degree of supramolecular environment, something that can be compensated by the design of the system, i.e., tuning properties such as the electrode/catalyst interface, local environment, electrode and device architecture to confer selectivity and activity benefits that can mimic some of the properties found within enzymes to improve their performance. These design considerations cross length scales, and must be tailored to the catalyst properties, with no universal solution and sits alongside efforts to develop improved bioinspired catalysts. However, there is much to learn by considering the full spectrum of catalysts and devices available as there are many parallels between them.

The state‐of‐the‐art development of CO_2_R electrolyzer seeks to incorporate crucial features found in enzyme structures on the molecular scale to control the availability of substrate, protons and the balance between hydrophobicity and hydrophilicity. While enzymes control these properties on nm lengthscales and electrolyzer properties are controlled on the μm to mm scale the parallels are evident. These similarities present an opportunity to draw insights from enzymes to improve the prospects of future CO_2_R electrolyzers.

## Conflict of interest

The authors declare no conflict of interest.

8

## Biographical Information

*Sam Cobb received his Ph.D. in Chemistry from the University of Warwick in 2019, supervised by Prof. Julie Macpherson, working on diamond as a material for electrochemical sensors. This was followed by Postdoctoral research at the University of Cambridge, where he was elected as a Research Fellow of Darwin College and awarded a Leverhulme Early Career Fellowship in 2021. This was further supported by an Isaac Newton Trust Fellowship and an Honorary Research Fellowship at the University of Warwick. In 2024 Sam will move to the University of Manchester to start his independent career as a Lecturer in Electrochemistry*.

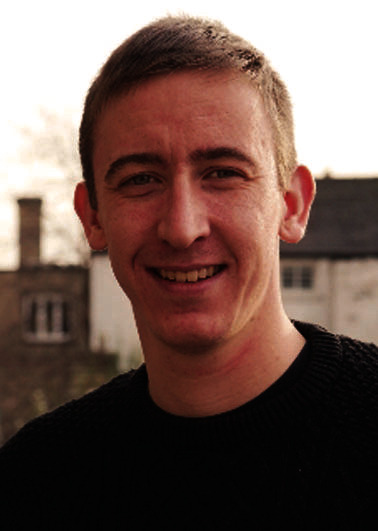

## Biographical Information

*Santiago Rodríguez Jiménez joined the Reisner lab as a Postdoctoral researcher in 2019, focusing on developing novel self‐assembled molecular systems for photocatalytic CO_2_ reduction. In 2021, he became a Marie Skłodowska‐Curie Fellow within the same laboratory and a NanoDTC Teaching Associate at the EPSRC Centre for Doctoral Training in Nanoscience and Nanotechnology. His current research, situated at the nexus of molecular materials and acetogenic bacteria, involves working on biohybrid systems capable of converting CO_2_ into valuable multicarbon products. Prior to joining the Reisner lab, he obtained his PhD in Chemistry from the University of Otago in 2017 under the supervision of Prof. Sally Brooker. His work there focused on designing molecular chemical sensors, and subsequently, as a postdoctoral researcher developing new hydrogen evolution molecular electrocatalysts*.

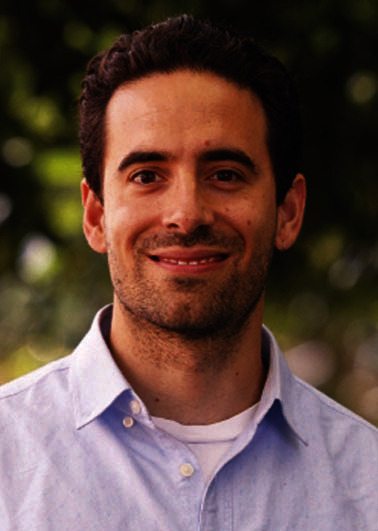

## Biographical Information

*Erwin Reisner is the Professor of Energy and Sustainability in the Yusuf Hamied Department of Chemistry at the University of Cambridge and a Fellow of St. John's College. His laboratory develops cross‐disciplinary concepts and technologies for the electrocatalytic and solar‐powered conversion of CO_2_, biomass and plastic waste streams into sustainable fuels and chemicals. His research on catalytic CO_2_ utilisation using synthetic and biological catalysts has been supported by the Christian Doppler Laboratory for Sustainable SynGas Chemistry (2012–2019), a European Research Council (ERC) Consolidator (2016–2023) and now by an ERC/UKRI Advanced Grant (2023–2028). He has received the 2023 Hughes Medal by the Royal Society for the development of solar chemical technologies, including the sustainable synthesis of fuels from CO_2_*.

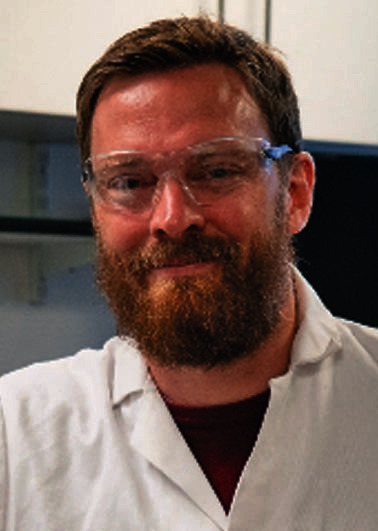

## Data Availability

Data sharing is not applicable to this article as no new data were created or analyzed in this study.
